# A simple preparation of very high methanol tolerant cathode electrocatalyst for direct methanol fuel cell based on polymer-coated carbon nanotube/platinum

**DOI:** 10.1038/srep12236

**Published:** 2015-07-20

**Authors:** Zehui Yang, Naotoshi Nakashima

**Affiliations:** 1 Department of Applied Chemistry, Graduate School of Engineering, Kyushu University, 744 Motooka Nishi-ku, Fukuoka 819-0395, Japan; 2 International Institute for Carbon-Neutral Energy Research (WPI-I2CNER), Kyushu University 744 Motooka Nishi-ku, Fukuoka 819-0395, Japan; 3 JST-CREST, 5 Sanbancho, Chiyoda-ku, Tokyo, 102-0075, Japan

## Abstract

The development of a durable and methanol tolerant electrocatalyst with a high oxygen reduction reaction activity is highly important for the cathode side of direct methanol fuel cells. Here, we describe a simple and novel methodology to fabricate a practically applicable electrocatalyst with a high methanol tolerance based on poly[2,2′-(2,6-pyridine)-5,5′-bibenzimidazole]-wrapped multi-walled carbon nanotubes, on which Pt nanoparticles have been deposited, then coated with poly(vinylphosphonic acid) (PVPA). The polymer coated electrocatalyst showed an ~3.3 times higher oxygen reduction reaction activity compared to that of the commercial CB/Pt and methanol tolerance in the presence of methanol to the electrolyte due to a 50% decreased methanol adsorption on the Pt after coating with the PVPA. Meanwhile, the peroxide generation of the PVPA coated electrocatalyst was as low as 0.8% with 2 M methanol added to the electrolyte, which was much lower than those of the non-PVPA-coated electrocatalyst (7.5%) and conventional CB/Pt (20.5%). Such a high methanol tolerance is very important for the design of a direct methanol fuel cell cathode electrocatalyst with a high performance.

Direct methanol fuel cells (DMFCs) are recognized as an ideal power source for mobile applications and have received considerable attention^1^,^2^,^3^,^4^. One main issue affecting the efficiency and power density of the DMFCs is methanol crossover, because methanol can easily go through a Nafion membrane and be oxidized at the cathode, poisoning the electrocatalyst and degrading the FC voltage and power density; especially when a high concentration of methanol is fed to the anode side^5^. Thus, the design of a new electrocatalyst with a methanol tolerance on the cathode side of the DMFCs is significantly required.

Recently, many studies have focused on the design and fabrication of a methanol tolerant electrocatalyst. The first trail used transition metals, such as Pd/Ag^6^,^7^, Ru/Se^8^,^9^, Pd/Co^10^,^11^, Ru/Mo/Se^12^,^13^, etc. Such electrocatalysts showed a high methanol tolerance due to the suppression of the methanol oxidation reaction (MOR). These transition metal electrocatalysts, however, are limited to practical DMFC applications because these transition metals show very low oxygen reduction reaction (ORR) activities compared to that of platinum (Pt), which is the most effective metal for the ORR^2^,^14^. Accordingly, the second trial used Pt alloys with transition metals, such as Pt/Au^15^,^16^, Pt/Pd/Cu^17^,^18^, Pt/Cr^19^,^20^, Pt/Ni^21^,^22^, Pt/Co^23^,^24^, etc., whereas, the durability of such electrocatalysts was low due to dissolution of the transition metals during real operating conditions^25^,^26^,^27^,^28^. Thus, the development of a durable and methanol tolerant electrocatalyst with a high ORR activity is still a significant and important challenge.

We here present the third trial for this issue (methanol tolerance) that shows a much higher performance than previous methods as well as an easy preparation based on a “polymer-coating” method. We have reported that the electrocatalyst coated with PVPA showed an enhancement in its fuel cell durability^29^,^30^ and blocked methanol absorption on the active Pt nanoparticles (Pt-NPs)^31^, which are important parameters for the cathode side of the DMFC. In this study, we describe the results that after coating with a polymer, the electrocatalyst showed a very high methanol tolerance.

## Results and Discussion

**Figure 1:**
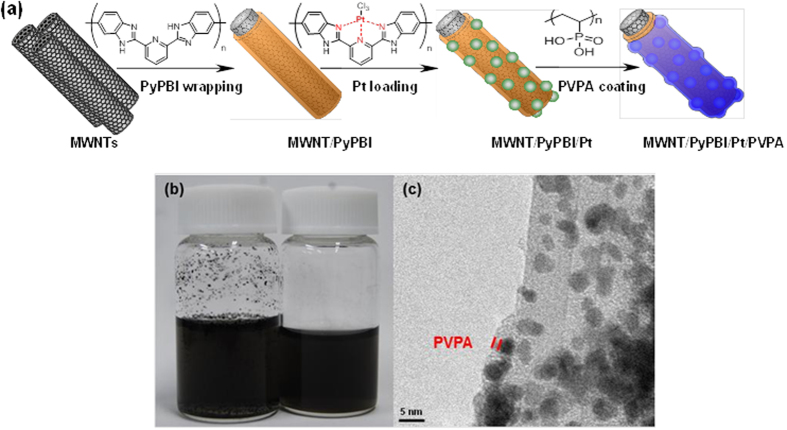
Procedure of the preparation and HR-TEM image of the MWNT/PyPBI/Pt/PVPA. (**a**) Schematic illustration for the preparation of the MWNT/PyPBI/Pt/PVPA. The chemical structures of PyPBI and PVPA are inserted in the illustration. (**b**) Photographs of the dispersity of the MWNT/PyPBI/Pt (left) and MWNT/PyPBI/Pt/PVPA in water. (**c**) HR-TEM image of the MWNT/PyPBI/Pt/PVPA.

The MWNT/PyPBI/Pt and MWNT/PyPBI/Pt/PVPA were prepared according to a previous method (Fig. 1a)^29^,^30^. The as-synthesized electrocatalysts were dispersed in water by sonication for 5 min to determine their dispersity in water. As shown in Fig. 1b, the MWNT/PyPBI/Pt/PVPA produced a homogenous solution due to the hydrophilic surfaces of the homogeneous coating of the PVPA layer, while the MWNT/PyPBI/Pt poorly dispersed in water due to the hydrophobic surfaces of the PyPBI. Also, the PVPA layer on the Pt-NPs determined by the HR-TEM shown in Fig. 1c was ~2 nm. The TEM images of the MWNT/PyPBI/Pt and MWNT/PyPBI/Pt/PVPA together with a conventional CB/Pt for comparison are shown in Fig. S1, from which their Pt diameters were determined to be 3.9 ± 0.2, 3.8 ± 0.2 and 3.8 ± 0.6 nm, respectively. The properties of these electrocatalysts were almost the same as those of the previous ones. Briefly, we observed two clear peaks at 71.4 and 75.0 eV that are attributed to the 4f_7/2_ and 4f_5/2_ of the metal Pt, respectively, and the N_1s_ peak was observed at ~400 eV in both MWNT/PyPBI/Pt and MWNT/PyPBI/Pt/PVPA that were derived from PyPBI (see Supplementary Information, Fig. S2). The typical P_2p_ peak due to the PVPA was observed at ~132 eV only in the MWNT/PyPBI/Pt/PVPA, suggesting that the PVPA successfully coated on the prepared electrocatalyst. The decrease in the density of the Pt_4f_ and N_1s_ peaks in the MWNT/PyPBI/Pt/PVPA also suggested that the Pt-NPs were covered by PVPA^31^. From the TGA curves (see Supplementary Information, Fig. S3), the Pt contents in the composites decreased from 47.3 wt% to 44.5 wt% due to the additional PVPA, which was 6.0 wt% in the MWNT/PyPBI/Pt/PVPA, that was similar to our previous report^29^, indicating a high controllability and reproducibility of our electrocatalyst, while the previous methanol tolerant electrocatalysts were difficult to synthesize due to their complex structure.

**Figure 2:**
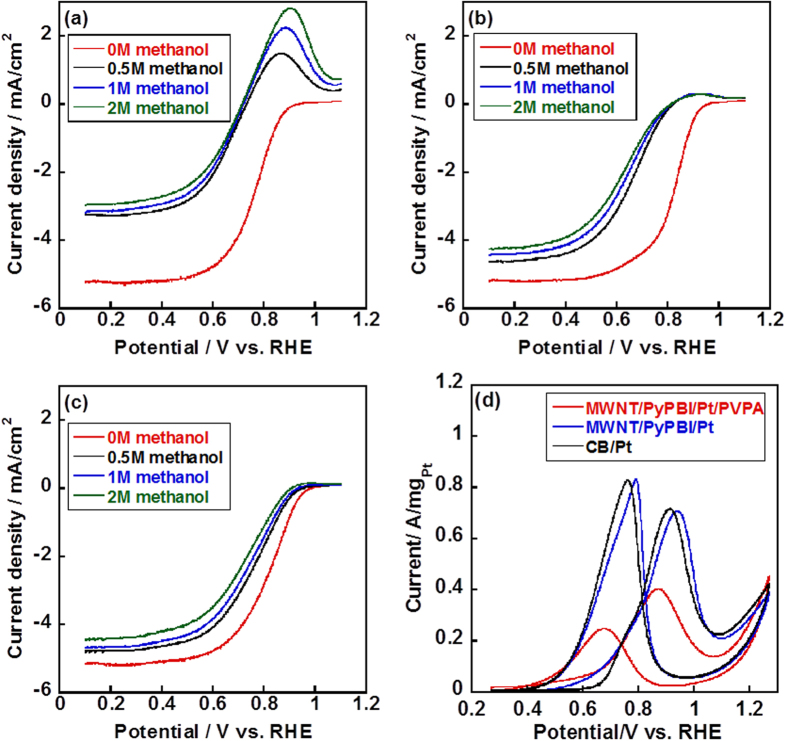
Electrochemical measurements of the conventional CB/Pt, MWNT/PyPBI/Pt and MWNT/PyPBI/Pt/PVA. ORR polarization curves for the CB/Pt (**a**), MWNT/PyPBI/Pt (**b**) and MWNT/PyPBI/Pt/PVPA (**c**) in O_2_-saturated 0.1 M HClO_4_ and varying concentrations of methanol at 25 °C, rotation rate of 1600 rpm, and sweep rate of 10 mV/s. In all figures, methanol concentrations are: 0 M (red), 0.5 M (black), 1 M (blue) and 2 M (green). (**d**) Methanol oxidation reaction (MOR) curves were recorded in 0.1 M HClO_4_ and 1 M methanol at a scan rate of 50 mV/s for the CB/Pt (black line), MWNT/PyPBI/Pt (blue line), and MWNT/PyPBI/Pt/PVPA (red line) before durability test.

The ORR is the cathodic reaction in an actual fuel cell^32^. During the fuel cell operation in the DMFCs, the methanol crossover is the most serious drawback since it lowers the voltage of the cells, leading to degradation of the FC performance. As shown in Fig. 2, the ORR was measured in the presence of a given concentration of methanol (for rotating disc current densities, see Supplementary Information, Fig. S4, 5 and 6). In the absence of methanol, the mass activities of the CB/Pt, MWNT/PyPBI/Pt and MWNT/PyPBI/Pt/PVPA calculated using the Levich-Koutecky equation, 1*/i* = 1*/i*_*k*_+1*/i*_*d*_(where *i* is the experimentally measured current, and *i*_*d*_ is the diffusion-limited current.), were 48.8, 187.0 and 157.7 mA/mg_Pt_ at 0.85 V *vs*. RHE, respectively^33^,^34^,^35^,^36^ The PVPA-coated electrocatalyst showed a slight decrease in the ORR activity by 15.6% compared to that of the MWNT/PyPBI/Pt due to the polymer-coating of the Pt-NPs. The specific activity was calculated by dividing the mass activity by the electrochemical surface area (ECSA). The ECSAs of the MWNT/PyPBI/Pt and MWNT/PyPBI/Pt/PVPA were 44.7 and 42.3 m^2^/g_Pt_, respectively, (see Supplementary Information, Fig. S7)^37^,^38^, and the Pt utilization efficiency of the MWNT/PyPBI/Pt was almost the same as described in our previous report^39^. The specific activities were 0.42 and 0.37 mA/cm^2^_Pt_ for the MWNT/PyPBI/Pt and MWNT/PyPBI/Pt/PVPA, respectively. These obtained values were much higher than that of the commercial CB/Pt (specific activity: 0.08 mA/cm^2^_Pt_, ECSA: 60.6 m^2^/g_Pt_) similar to previous reports^40^,^41^. Meanwhile, the diffusion-limited current density of the PVPA-coated electrocatalyst (−5.2 mA/cm^2^, see red line in Fig. 2c) was almost identical to that of the MWNT/PyPBI/Pt (−5.3 mA/cm^2^, see red line in Fig. 2b), suggesting that the PVPA coating showed a negligible effect on the O_2_ accessibility^42^. For the MWNT/PyPBI/Pt, we observed small methanol oxidation peak at 0.9 V *vs*. RHE in the presence of methanol in the electrolyte (see Fig. 2b). On the contrary, almost no such peak was recognized for the MWNT/PyPBI/Pt/PVPA, indicating the suppression of the methanol oxidation due to effect of methanol absorption caused by the polymer coating^31^,^43^.

It should be noted that an obvious methanol oxidation peak was observed for the conventional CB/Pt (see Fig. 2a) in the presence of methanol in the electrolyte due to the high ECSA value (see Supplementary Information, Fig. S7). The MOR peak showed a positive shift and the peak current increased with the increased methanol concentration as shown in Fig. 2a. The half-wave potentials (*E*_*1/2*_) evaluated from the ORR activity of the CB/Pt, MWNT/PyPBI/Pt and MWNT/PyPBI/Pt/PVPA are shown in Table 1^34^,^44^,^45^,^46^,^47^, in which, in the absence of the methanol, the value of the MWNT/PyPBI/Pt was the highest, which is consistent with the analyses of the mass and specific activities. With the increase in the methanol concentration, the *E*_1/2_ of the three electrocatalysts decreased due to the coverage of CO on the Pt-NPs generated during the methanol oxidation reaction. However, in the presence of a high concentration of methanol, the *E*_1/2_ of the CB/Pt and MWNT/PyPBI/Pt sharply decreased by 150 mV and 200 mV (see Table 1), respectively, while for the MWNT/PyPBI/Pt/PVPA, the value decreased only by 80 mV (see Table 1), suggesting that after coating with the PVPA, the presence of methanol showed the lowest effect on the ORR activity. Besides, the *E*_1/2_ was higher than those of the CB/Pt and MWNT/PyPBI/Pt at the same methanol concentration.

**Figure 3:**
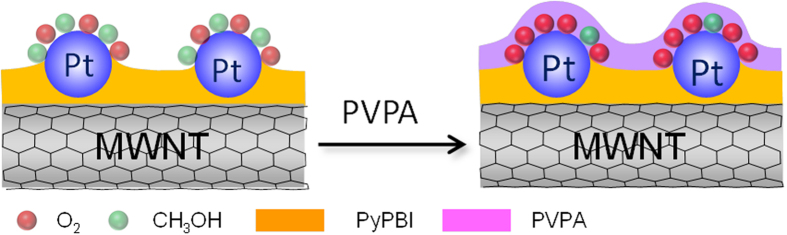
Mechanism of high methanol tolerance for the MWNT/PyPBI/Pt/PVPA. Schematic illustration showing a low methanol tolerance of the MWNT/PyPBI/Pt (left) and high methanol tolerance (right) of the MWNT/PyPBI/Pt/PVPA.

Compared to the MWNT/PyPBI/Pt/PVPA (see red and green lines in Fig. 2c, 1.0 mA/cm^2^), the ORR curves of the CB/Pt (see red and green lines in Fig. 2a) and MWNT/PyPBI/Pt (see red and green lines in Fig. 2b) showed high negative shifts (3.0 and 1.9 mA/cm^2^ at 0.85 V *vs*. RHE for the CB/Pt and MWNT/PyPBI/Pt, respectively) when 2 M methanol was added to the electrolyte, indicating that the CB/Pt and MWNT/PyPBI/Pt heavily suffered from methanol poisoning^6^. Also, the diffusion limited current density of the MWNT/PyPBI/Pt/PVPA showed a decrease only by 0.7 mA/cm^2^ (see Fig. 2c) after adding 2 M methanol to the electrolyte, indicating a slight effect on the O_2_ accessibility due to the prevention of methanol absorption on the Pt-NPs by the PVPA coating, while the MWNT/PyPBI/Pt and CB/Pt showed a 1.0 and 2.3 mA/cm^2^-loss (see Fig. 2a,2b) in the diffusion-limited current densities, respectively. Watanabe *et al.* pointed out that during the ORR, the oxygen molecules were supplied by spherical diffusion to the individual Pt-NPs^48^,^49^. The present study indicated that after coating with the PVPA, the methanol absorption was partly blocked as shown in Fig. 2d in which the mass current density of the MWNT/PyPBI/Pt/PVPA decreased by ~50% compared to that of the MWNT/PyPBI/Pt due to the coverage of the Pt-NP surfaces. The isolation of the electrocatalyst from the methanol is highly important for prepare a methanol-tolerant electrocatalyst. Meanwhile, the PVPA showed almost no effect on the O_2_ diffusion due to the smaller size of the oxygen molecule and easy diffusion. Thus, the oxygen occupied the areas (radial diffusion fields) of the Pt-NPs and was reduced by the Pt-NPs in the MWNT/PyPBI/Pt/PVPA. As schematically shown in Fig. 3, methanol is predicted to be difficult to absorb on the active Pt-NP surfaces, meanwhile, the possibility of methanol absorption was almost comparable to oxygen for the MWNT/PyPBI/Pt and conventional CB/Pt.

**Figure 4:**
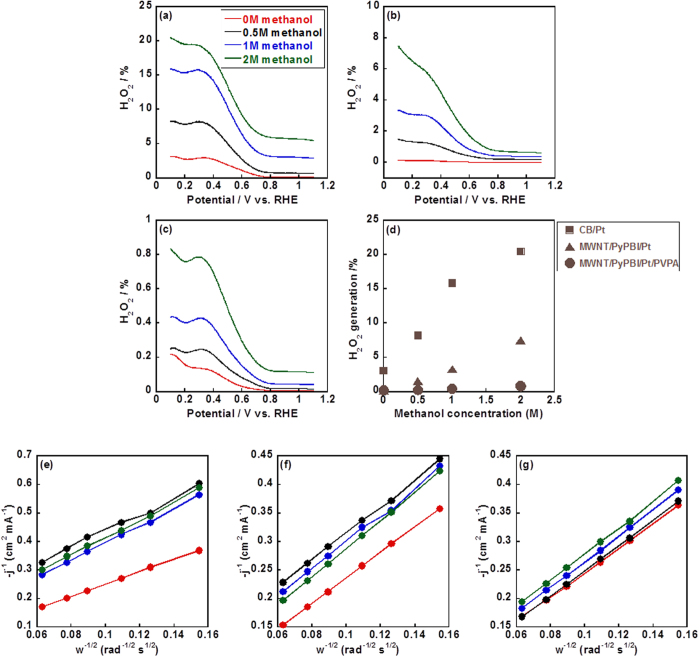
Analyses of H_2_O_2_ generations and transferred electron. Peroxide generations of CB/Pt (**a**) MWNT/PyPBI/Pt (**b**) and MWNT/PyPBI/Pt/PVPA (**c**) from the ORR detected at the ring electrode in O_2_-saturated 0.1 M HClO_4_ and varying concentration of methanol at the sweep rate of 10 mV/s, and rotation rate of 1600 rpm. (d) H_2_O_2_ generations of the CB/Pt (■), MWNT/PyPBI/Pt (▲) and MWNTs/PyPBI/Pt/PVPA (•) as a function of methanol concentration in the electrolyte. Levich-Koutecky plots from the ORR at 0.43 V *vs*. RHE for CB/Pt (**e**), MWNT/PyPBI/Pt (**f**) and MWNT/PyPBI/Pt/PVPA (**g**). In figures (a, b, c, e, f, g), methanol concentrations are: 0 M (red), 0.5 M (black), 1 M (blue) and 2 M (green).

Peroxide (H_2_O_2_) is an unwanted product during the ORR since it degrades the FC durability *via* corrosion of the carbon materials^50^. As shown in Fig. 4(a–c), the H_2_O_2_ generations of the CB/Pt, MWNT/PyPBI/Pt and MWNT/PyPBI/Pt/PVPA were calculated using the equation, %H_2_O_2_ = 200*I*_*R*_/(*NI*_*D*_+*I*_*R*_), where *I*_*R*_ and *I*_*D*_ are the ring and disk currents obtained from the ORR measurement, respectively, and *N* is the collection efficiency, which corresponds to the *I*_*R*_*/I*_*D*_ ratio (0.404) measured in N_2_-saturated 0.1 M KCl and 1 mM K_3_Fe(CN)_6_^31^. These values were accelerated with the increase in the methanol concentrations as shown in Fig. 4d, which was attributed to the decrease in the transferred electrons as shown in Fig. 4(e–g) and Table 2.

In the absence of methanol, the H_2_O_2_ generations of the three electrocatalysts were very low due to the four electrons transferred during the ORR. However, under the high methanol concentration of 2 M, the H_2_O_2_ generations of the CB/Pt and MWNT/PyPBI/Pt dramatically increased to 20.5% and 7.5%, which were much higher than that (0.8%) for the MWNT/PyPBI/Pt/PVPA. As is well known, the oxygen was first reduced to H_2_O_2_, then the H_2_O_2_ was further reduced to H_2_O as the ideal product. While the high methanol concentration led to the heavy poisoning of the Pt-NPs, thus the intermediate (H_2_O_2_) absorbed on the Pt-NPs surface cannot be completely reduced to H_2_O. The lower H_2_O_2_ generation on MWNT/PyPBI/Pt/PVPA would be due to the PVPA polymer layer that prevents the H_2_O_2_ detaching from the electrocatalyst, which is important for the reduction to H_2_O on the Pt-NPs, while the H_2_O_2_ generated on CB/Pt and MWNT/PyPBI/Pt would be easily released from the surfaces of the electrocatalysts^5^. Such higher H_2_O_2_ generations for the CB/Pt and MWNT/PyPBI/Pt should accelerate the corrosion of the carbon supporting materials, resulting in the decreased Pt-NPs stability. In sharp contrast, the polymer coated electrocatalyst, MWNT/PyPBI/Pt/PVPA, showed a high methanol tolerance and low H_2_O_2_ generation and 3.8 electrons were transferred during the ORR, which is close to the ideal ORR, suggesting that the MWNT/PyPBI/Pt/PVPA is highly important for practical use as a cathode electrocatalyst for the DMFCs. Accordingly, a high concentration of methanol can be fed to the anode side to enhance the FC performance.

**Figure 5:**
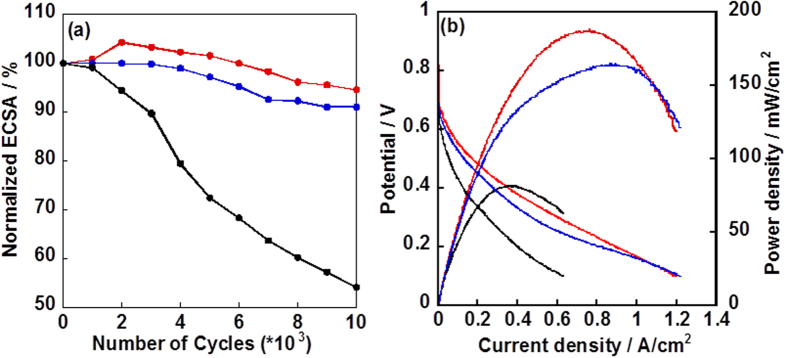
Durability test and Single cell performance of three electrocatalysts. (**a**) Plots of normalized ECSAs of the CB/Pt (black line), MWNT/PyPBI/Pt (Blue line) and MWNT/PyPBI/Pt/PVPA (red line) as a function of the numbers of potential cycles from 1.0 to 1.5 V *vs*. RHE. (**b**) Polarization I-V and power density curves of MEAs fabricated from the CB/Pt (black line), MWNT/PyPBI/Pt (Blue line) and MWNT/PyPBI/Pt/PVPA (red line) under 70 ^o^C with 8 M methanol (9 mL/min) and 100%RH humidified air (200 mL/min) for anode and cathode, respectively.

In order to study the durability of the electrocatalysts, we tested the durability of three electrocatalysts according to the protocol from the Fuel Cell Conference of Japan (FCCJ) in which the carbon corrosion (C+2H_2_O→CO_2_+4H^+^+4e^–^, 0.207 V *vs*. RHE) is accelerated. The loss in carbon supporting material leads to degradation of fuel cell performance due to the loss of the Pt-NPs. As shown in Fig. 5a, the ECSA of the CB/Pt lost ~46% after 10,000 cycles from/to 1.0 to/from 1.5 V *vs*. RHE is due to the lower carbon corrosion resistance. The MWNT/PyPBI/Pt showed an ~10% loss in the ECSA after the potential cycling, while the MWNT/PyPBI/Pt/PVPA showed the highest durability among the three electrocatalysts (~6% loss in the ECSA) due to the high resistance towards carbon corrosion and the PVPA coating, which also reduced the carbon corrosion and Pt agglomeration. The membrane electrode assemblies (MEAs) were fabricated for practical application in DMFC system. The polarization curves of the MEAs measured under 100% relative humidity (RH) at 70 ^o^C are shown in Fig. 5b. Before applying a current to the single cell, the open circuit voltages (OCVs) were 0.66, 0.69 and 0.82 V for the CB/Pt, MWNT/PyPBI/Pt and MWNT/PyPBI/Pt/PVPA, respectively, in which the observed higher OCV of the MWNT/PyPBI/Pt/PVPA suggested a higher methanol tolerance. Even though the MWNTs were coated by PyPBI and PVPA, little or no decrease in the electronic conductivity was observed due to the thin layer of the PyPBI (1–2 nm)^51^,^52^. The maximum power density of the MEA fabricated from MWNT/PyPBI/Pt/PVPA was 187 mW/cm^2^ which was ~2.3 times higher than that of the commercial CB/Pt (81 mW/cm^2^). The MEA fabricated from MWNT/PyPBI/Pt was 165 mW/cm^2^ which was lower than that of the MWNT/PyPBI/Pt/PVPA due to the homogeneous PVPA coating that acted as a proton conductor during the fuel cell measurement.

## Conclusions

In conclusion, we have demonstrated that after a simple coating with PVPA, the obtained MWNT/PyPBI/Pt/PVPA functions as a cathode electrocatalyst of the DMFCs with a high performance. This electrocatalyst has advantages compared to the previously reported electrocatalysts described in the Introduction since its performance is very high and the preparation method is very simple and easy. Such a high performance is due to the coated PVPA layer that blocks the methanol absorption and has no effect on the O_2_ diffusion. As a result, the H_2_O_2_ generation was highly restricted (only 0.8% even in the presence of 2 M methanol in the electrolyte, which was much lower than those of the MWNT/PyPBI/Pt (7.5%) and CB/Pt (20.5%). Meanwhile, the power density of the MWNT/PyPBI/Pt/PVPA was ~2.3 times higher than that of the commercial CB/Pt (81 mW/cm^2^).

The present study provides a new strategy for the design a practically applicable electrocatalyst as a DMFC cathode with a high methanol tolerance.

## Method

### Materials

H_2_PtCl_6_·6H_2_O, 2-propanol, *N,N*-dimethylacetamide (DMAc), ethylene glycol (EG) and poly(vinylphosphonic acid) (PVPA, 30 wt%) were purchased from Wako Pure Chemical Industry, Ltd. Commercial CB/Pt (Pt amount: 37.9 wt%) was obtained from Tanaka Kikinzuku Kogyo KK. Perchloric acid (70%) was purchased from EMD Millipore Chemical Co., Ltd. Methanol was purchased from Kanto Chemical Co., Inc. All the chemicals were used as received without any purification. The MWNTs with a ~20 nm diameter were kindly provided by Nikkiso Co., Ltd.

### Synthesis of MWNT/PyPBI/Pt/PVPA

MWNTs (10 mg) dispersed in DMAc (20 mL) by sonication for 1 h and PyPBI (5 mg) dissolved in DMAc (10 mL) were mixed and ultrasonicated for 2 h, then filtered to obtain a solid (MWNT/PyPBI), which was dried overnight under vacuum at 80 ^o^C. The deposition of Pt nanoparticles (Pt-NPs) was carried out by the reduction of H_2_PtCl_6_·6H_2_O in EG aqueous solution (EG:H_2_O = 3/2,*v/v*). First, 10 mg of MWNT/PyPBI was dissolved in a 30 mL EG aqueous solution to which 24 mg of H_2_PtCl_6_·6H_2_O in EG (20 mL) was added. The mixture was then refluxed at 140 ^o^C for 6 h under N_2_ atmosphere. The catalysts were collected by filtration, then dried overnight in oven at 80 ^o^C to remove the remained solvent. MWNT/PyPBI/Pt (10 mg) dissolved in a 10 mL EG aqueous solution (*v/v* = 3:2) by sonication for 5 min and 30 wt% PVPA aqueous solution (1 mL) was mixed and sonicated with a bath-type sonicator for 1 h. The dispersion was then filtered with a 0.1 μm PTFE filtrate paper, and washed several times by Milli-Q water to obtain MWNT/PyPBI/Pt/PVPA, which was dried overnight under vacuum at 60 ^o^C.

### Gas diffusion electrode (GDE) fabrication

The GDE was fabricated as follows. The electrocatalyst was dispersed in 50 mL of a 2-propanol aqueous solution by sonication for 1 h, then filtrered using a gas diffusion layer (GDL) as a filter paper. The Pt loading amount on the GDL was controlled at 2 mg/cm^2^. The obtained GDE was dried overnight under vacuum at room temperature to remove any residual solvent.

### Membrane electrode assembly (MEA) fabrication

The MEA was prepared by hot pressing the prepared GDE and a Nafion 117 membrane. The active area of the MEA was 1 cm^2^.

### Characterization

The X-ray photoelectron spectroscopy (XPS) spectra were measured using an AXIS-ULTRA^DLD^ (Shimadzu) instrument. The TGA measurements were conducted using an EXSTAR 6000, Seiko, Inc., instrument at the heating rate of 5 ºC/min under 100 mL/min of air. The TEM micrographs were measured using a JEM-2010 (JEOL, acceleration voltage of 120 kV) electron microscope, in which a copper grid with a carbon support (Okenshoji) was used.

### Electrochemical measurement

The electrochemical measurements were performed using a rotating ring disk electrode attached to an RRDE-3 (Bioanalytical Systems, Inc.) with a conventional three-electrode configuration in a vessel at room temperature. A glassy carbon electrode (GCE) with a geometric surface area of 0.196 cm^2^ was used as the working electrode. A Pt wire and an Ag/AgCl were used as the counter and reference electrodes, respectively. The potential of the electrode was controlled by an ALS-Model DY2323 (BAS) potentiostat. The electrocatalyst suspension was typically prepared as follows. The electrocatalyst (1.0 mg) was ultrasonically dispersed in an 80% aqueous EG solution (2.0 mL) to form a homogeneous dispersion. A portion of the dispersion was then cast on a GCE to provide an electrocatalyst film, which was air-dried on which the Pt-NPs (14 μg/cm^2^) were deposited. The CVs of the electrocatalysts were carried out at a scan rate of 50 mV/s in N_2_-saturated 0.1 M HClO_4_ solutions to determine the ECSA values. The oxygen reduction reaction (ORR) was conducted in O_2_-saturated 0.1 M HClO_4_ and the given concentration of methanol using a rotation ring disk electrode (RRDE) system (BAS, Inc).

### MOR evaluation

The methanol oxidation reaction (MOR) was evaluated before and after the durability test using N_2_-saturated 1 M-methanol and 0.1 M HClO_4_ at the scan rate of 50 mV/s at room temperature without rotation. The electrode was the same as that used for the ECSA measurements. The Pt loading amounts were controlled at 14 μg/cm^2^. Before the MOR measurements, 50 cycles were carried out to activate the electrocatalysts.

### Durability testing

The carbon corrosion was severely tested using the protocol of the Fuel Cell Commercialization Conference of Japan (FCCJ)^53^ (measured in N_2_-saturated 0.1 M HClO_4_ at room temperature without rotation), in which the potential was maintained at 1.0 V *vs*. RHE for 30 s, then applied to 1.5 V *vs*. RHE at the scan speed of 0.5 V/s followed by a potential-return to 1 V *vs*. RHE. This procedure was cycled, and after every 1000 cycles, ECSA measurements were carried out (see Supplementary Information, Fig. S8).

### Fuel cell testing

The FC performance of the assembled MEAs was evaluated at 70 °C using a computer-controlled fuel cell test system (Model 890e, Scribner Associate, Inc.). The polarization and power density curves were obtained at the atmospheric pressure by flowing 8 M methanol (flow rate = 9 mL/min) and 100% relative humidified air (flow rate = 200 mL/min) to the anode and cathode, respectively.

## Additional Information

**How to cite this article**: Yang, Z. and Nakashima, N. A simple preparation of very high methanol tolerant cathode electrocatalyst for direct methanol fuel cell based on polymer-coated carbon nanotube/platinum. *Sci. Rep.* **5**, 12236; doi: 10.1038/srep12236 (2015).

## Supplementary Material

Supplementary Information

## Figures and Tables

**Table 1:** Half-wave potential (*E*_*1/2*_) of the three electrocatalysts during the ORR measurements in the presence of the given methanol concentrations.

Electrocatalyst	0 M	0.5 M	1 M	2 M
CB/Pt	0.76 V	0.63 V	0.62 V	0.61 V
MWNT/PyPBI/Pt	0.82 V	0.66 V	0.63 V	0.62 V
MWNT/PyPBI/Pt/PVPA	0.80 V	0.75 V	0.74 V	0.72 V

**Table 2:** Number of the transferred electrons of three electrocatalysts during the ORR measurement in the presence of different concentrations of methanol.

Electrocatalyst	0 M	0.5 M	1 M	2 M
CB/Pt	4.0	3.0	2.9	2.8
MWNT/PyPBI/Pt	3.9	3.7	3.6	3.5
MWNT/PyPBI/Pt/PVPA	4.0	3.9	3.8	3.8

## References

[b1] Zhao X. *et al.* Recent advances in catalysts for direct methanol fuel cells. Energy Environ Sci. 4, 2736–2753 (2011).

[b2] Gao M. R. *et al.* A methanol-tolerant Pt/CoSe2 nanobelt cathode catalyst for direct methanol fuel cells. Angew Chem Int Ed. 50, 4905–4908 (2011). 10.1002/anie.201007036 21438105

[b3] Wen Z., Liu J. & Li J. Core/shell Pt/C nanoparticles embedded in mesoporous carbon as a methanol-tolerant cathode catalyst in direct methanol fuel cells. Adv Mater 20, 743–747 (2008).

[b4] Koenigsmann C., Wong S. S. One-dimensional noble metal electrocatalysts: A promising structural paradigm for direct methanol fuel cells. Energy Environ Sci. 4, 1161–1176 (2011).

[b5] Franceschini E. A. *et al.* Mesoporous Pt electrocatalyst for methanol tolerant cathodes of DMFC. Electrochim Acta 71, 173–180 (2012).

[b6] Liu M., Lu Y. & Chen W. PdAg Nanorings Supported on Graphene Nanosheets: Highly Methanol-Tolerant Cathode Electrocatalyst for Alkaline Fuel Cells. Adv Funct Mater 23, 1289–1296 (2013).

[b7] Sekol R. C. *et al.* Silver palladium core-shell electrocatalyst supported on MWNTs for ORR in alkaline media. Appl Catal B 138-139, 285–293 (2013).

[b8] Colmenares L., Jusys Z. & Behm R. J. Activity, selectivity, and methanol tolerance of Se-modified Ru/C cathode catalysts. J Phys Chem C 111, 1273–1283 (2007).

[b9] Choi J. H., *et al.* Se-modified Ru nanoparticles as ORR catalysts: Part 2: Evaluation for use as DMFC cathodes. J Electroanal Chem. 662, 267–273 (2011).

[b10] Gharibi H., Golmohammadi F., Kheirmand M. Fabrication of MEA based on optimum amount of Co in PdxCo/C alloy nanoparticles as a new cathode for oxygen reduction reaction in passive direct methanol fuel cells. Electrochim Acta 89, 212–221 (2013).

[b11] Li X. *et al.* Low temperature preparation of carbon-supported Pd-Co alloy electrocatalysts for methanol-tolerant oxygen reduction reaction. Electrochim Acta. 53, 6662–6667 (2008).

[b12] Yu J. S., Kim M. S. & Kim J. H. Combinatorial discovery of new methanol-tolerant non-noble metal cathode electrocatalysts for direct methanol fuel cells. Phys Chem Chem Phys. 12, 15274–15281 (2010). 20953489 10.1039/c0cp00767f

[b13] Schmidt T. J. *et al.* Oxygen reduction on Ru1.92Mo0.08SeO4, Ru/carbon, and Pt/carbon in pure and methanol-containing electrolytes. J Electrochem Soc. 147, 2620–2624 (2000).

[b14] Selvarani G. *et al.* Carbon-supported Pt- TiO2 as a methanol-tolerant oxygen-reduction catalyst for DMFCs. J Electrochem Soc 156, B1354–B1360 (2009).

[b15] Selvarani G., *et al.* A methanol-tolerant carbon-supported pt-au alloy cathode catalyst for direct methanol fuel cells and its evaluation by DFT. J Phys Chem C 113, 7461–7468 (2009).

[b16] Wang J. *et al.* Carbon nanotubes supported Pt–Au catalysts for methanol-tolerant oxygen reduction reaction: A comparison between Pt/Au and PtAu nanoparticles. J Power Sources 194, 668–673 (2009).

[b17] Cochell T., Li W. & Manthiram A. Effects of Pt coverage in Pt@PdCu5/C core-shell electrocatalysts on the oxygen reduction reaction and methanol tolerance. J Phys Chem C 117, 3865–3873 (2013).

[b18] Nishanth K. G., Sridhar P., Pitchumani S., Shukla A. K. A DMFC with methanol-tolerant-carbon-supported-Pt-Pd-alloy cathode. J Electrochem Soc. 158, B871–B876 (2011).

[b19] Yang H., Alonso-Vante N., Léger J. M. & Lamy C. Tailoring, Structure, and Activity of Carbon-Supported Nanosized Pt-Cr Alloy Electrocatalysts for Oxygen Reduction in Pure and Methanol-Containing Electrolytes. J Phys Chem B 108, 1938–1947 (2004).

[b20] Maillard F., Martin M., Gloaguen F. & Léger J. M. Oxygen electroreduction on carbon-supported platinum catalysts. Particle-size effect on the tolerance to methanol competition. Electrochim Acta 47, 3431–3440 (2002).

[b21] Drillet J. F. *et al.* Oxygen reduction at Pt and Pt70Ni30 in H2SO4/CH3OH solution. Electrochim Acta 47, 1983–1988 (2002).

[b22] Yang H. *et al.* Methanol tolerant oxygen reduction on carbon-supported Pt-Ni alloy nanoparticles. J Electroanal Chem. 576, 305–313 (2005).

[b23] Lima F. H. B. *et al.* Pt-Co/C nanoparticles as electrocatalysts for oxygen reduction in H2SO4 and H2SO4/CH3OH electrolytes. Electrochim Acta 52, 385–393 (2006).

[b24] Salgado J. R. C., Antolini E., Gonzalez E. R. Carbon supported Pt–Co alloys as methanol-resistant oxygen-reduction electrocatalysts for direct methanol fuel cells. Appl Catal B 57, 283–290 (2005).

[b25] Stephens I. E. L. *et al.* Understanding the electrocatalysis of oxygen reduction on platinum and its alloys. Energy Environ Sci. 5, 6744–6762 (2012).

[b26] Hwang S. J. *et al.* Role of Electronic Perturbation in Stability and Activity of Pt-Based Alloy Nanocatalysts for Oxygen Reduction. J Am Chem Soc. 134, 19508–19511 (2012). 23131009 10.1021/ja307951y

[b27] Stamenkovic V. R. *et al.* Effect of Surface Composition on Electronic Structure, Stability, and Electrocatalytic Properties of Pt-Transition Metal Alloys: Pt-Skin versus Pt-Skeleton Surfaces. J Am Chem Soc. 128, 8813–8819 (2006). 16819874 10.1021/ja0600476

[b28] Wang S., Cochell T. & Manthiram A. Boron-doped carbon nanotube-supported Pt nanoparticles with improved CO tolerance for methanol electro-oxidation. Phys Chem Chem Phys. 14, 13910–13913 (2012). 22990139 10.1039/c2cp42414b

[b29] Berber M. R., Fujigaya T., Sasaki K. & Nakashima N. Remarkably Durable High Temperature Polymer Electrolyte Fuel Cell Based on Poly(vinylphosphonic acid)-doped Polybenzimidazole. Sci Rep. 3, art number 1763 (2013).

[b30] Berber M. R., Fujigaya T. & Nakashima N. High-temperature polymer electrolyte fuel cell using poly(vinylphosphonic acid) as an electrolyte shows a remarkable durability. ChemCatChem. 6, 567–571 (2014).

[b31] Yang Z., Berber M. R., Nakashima N. A polymer-coated carbon black-based fuel cell electrocatalyst with high CO-tolerance and durability in direct methanol oxidation. J Mater Chem A 2, 18875–18880 (2014).

[b32] Wang J. X., Markovic N. M., Adzic R. R. Kinetic Analysis of Oxygen Reduction on Pt(111) in Acid Solutions: Intrinsic Kinetic Parameters and Anion Adsorption Effects. J Phys Chem B 108, 4127–4133 (2004).

[b33] Li Y. *et al.* Stabilization of high-performance oxygen reduction reaction Pt electrocatalyst supported on reduced graphene oxide/carbon black composite. J Am Chem Soc. 134, 12326–12329 (2012). 22783832 10.1021/ja3031449

[b34] Wang D. *et al.* Structurally ordered intermetallic platinum-cobalt core-shell nanoparticles with enhanced activity and stability as oxygen reduction electrocatalysts. Nat Mater 12, 81–87 (2013). 23104154 10.1038/nmat3458

[b35] He G. *et al.* Oxygen Reduction Catalyzed by Platinum Nanoparticles Supported on Graphene Quantum Dots. ACS Catal 3, 831–838 (2013).

[b36] Guo S., Sun S. FePt Nanoparticles Assembled on Graphene as Enhanced Catalyst for Oxygen Reduction Reaction. J Am Chem Soc. 134, 2492–2495 (2012). 22279956 10.1021/ja2104334

[b37] Cochell T., Manthiram A. Pt@PdxCuy/C Core–Shell Electrocatalysts for Oxygen Reduction Reaction in Fuel Cells. Langmuir 28, 1579–1587 (2011). 22149212 10.1021/la202610z

[b38] Lim B. *et al.* Pd-Pt Bimetallic Nanodendrites with High Activity for Oxygen Reduction. Science 324, 1302–1305 (2009). 19443738 10.1126/science.1170377

[b39] Okamoto M., Fujigaya T., Nakashima N. Design of an assembly of poly(benzimidazole), carbon nanotubes, and Pt nanoparticles for a fuel-cell electrocatalyst with an ideal interfacial nanostructure. Small 5, 735–740 (2009). 19263429 10.1002/smll.200801742

[b40] Beak S., Jung D., Nahm K. S., Kim P. Preparation of highly dispersed Pt on TiO2-modified carbon for the application to oxygen reduction reaction. Catal Lett. 134, 288–294 (2010).

[b41] Jang J.-H. *et al.* One-pot synthesis of core-shell-like Pt3Co nanoparticle electrocatalyst with Pt-enriched surface for oxygen reduction reaction in fuel cells. Energy Environ Sci. 4, 4947–4953 (2011).

[b42] Matsumoto K., Fujigaya T., Yanagi H. & Nakashima N. Very High Performance Alkali Anion-Exchange Membrane Fuel Cells. Adv Funct Mater 21, 1089–1094 (2011).

[b43] Fujigaya T. *et al.* Interfacial engineering of platinum catalysts for fuel cells: Methanol oxidation is dramatically improved by polymer coating on a platinum catalyst. ChemCatChem. 5, 1701–1704 (2013).

[b44] Guo S., Zhang S., Sun S. Tuning nanoparticle catalysis for the oxygen reduction reaction. Angew Chem Int Ed. 52, 8526–8544 (2013). 10.1002/anie.201207186 23775769

[b45] Wang D. *et al.* Tuning oxygen reduction reaction activity via controllable dealloying: A model study of ordered Cu 3Pt/C intermetallic nanocatalysts. Nano Lett. 12, 5230–5238 (2012). 22954373 10.1021/nl302404g

[b46] Alia S. M. *et al.* Porous Platinum Nanotubes for Oxygen Reduction and Methanol Oxidation Reactions. Adv Funct Mater 20, 3742–3746 (2010).

[b47] Wu G., More K. L., Johnston C. M., Zelenay P. High-Performance Electrocatalysts for Oxygen Reduction Derived from Polyaniline, Iron, and Cobalt. Science 332, 443–447 (2011). 21512028 10.1126/science.1200832

[b48] Lee M., *et al.* Durability of Pt/graphitized carbon catalyst prepared by the nanocapsule method for the start/stop operating condition of polymer electrolyte fuel cells. Electrochemistry 79, 381–387 (2011).

[b49] Yano H., *et al.* Durability of Pt/graphitized carbon catalysts for the oxygen reduction reaction prepared by the nanocapsule method. Phys Chem Chem Phys 12, 3806–3814 (2010). 20358074 10.1039/b923460h

[b50] Yuan X., Ding X. L., Wang C. Y., Ma Z. F. Use of polypyrrole in catalysts for low temperature fuel cells. Energy Environ Sci 6, 1105–1124 (2013).

[b51] Fujigaya T., Okamoto M., Nakashima N. Design of an assembly of pyridine-containing polybenzimidazole, carbon nanotubes and Pt nanoparticles for a fuel cell electrocatalyst with a high electrochemically active surface area. Carbon 47, 3227–3232 (2009).

[b52] Yang Z., Moriguchi I., Nakashima N. Durable Pt Electrocatalyst Supported on a 3D Nanoporous Carbon Shows High Performance in a High-Temperature Polymer Electrolyte Fuel Cell. ACS Appl Mater Interfaces 7, 9800–9806 (2015). 25902007 10.1021/acsami.5b01724

[b53] Ohma A., *et al.* Membrane and catalyst performance targets for automotive fuel cells by FCCJ membrane, catalyst, MEA WG. ECS Trans 41, 775–784 (2011).

