# Supplementary material for: A simple preparation of very high methanol tolerant cathode electrocatalyst for direct methanol fuel cell based on polymer-coated carbon nanotube/platinum
Source: Sci Rep. 2015 Jul 20;5:12236. doi: 10.1038/srep12236 (PMC4507447; doi:10.1038/srep12236)
Supplement: Supplementary Information [file srep12236-s1.pdf]

Supplementary Information

A simple preparation of a very high methanol tolerance cathode electrocatalyst for direct methanol fuel cell based on polymer-coated carbon nanotube/platinum

*Zehui Yang and Naotoshi Nakashima**

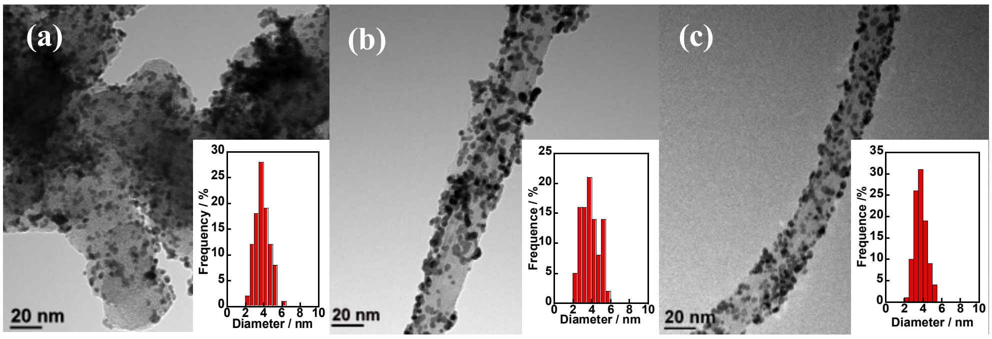

Figure S1| TEM images of the CB/Pt (a), MWNT/PyPBI/Pt (b) and MWNT/PyPBI/Pt/PVPA (c). Histograms of their particle size distribution (100 particles) are shown in the inset.

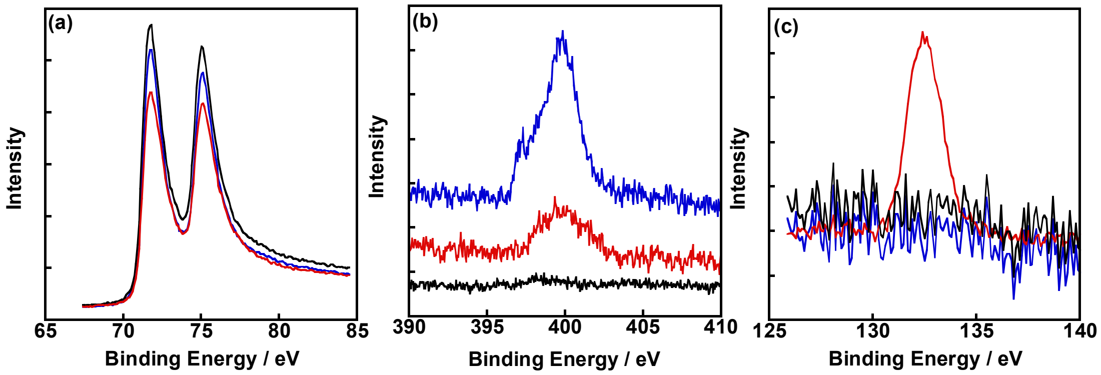

Figure S2| XPS narrow scans in the regions of Pt_{4f} (a), N_{1s} (b) and P_{2p} (c) of the CB/Pt (black), MWNT/PyPBI/Pt (blue) and MWNT/PyPBI/Pt/PVPA (red).

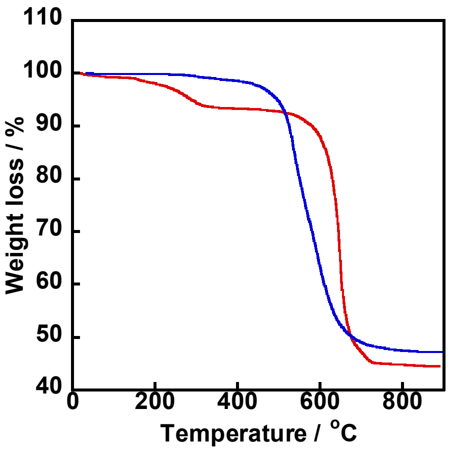

Figure S3| TGA curves of the MWNT/PyPBI/Pt (blue) and MWNT/PyPBI/Pt/PVPA (red).

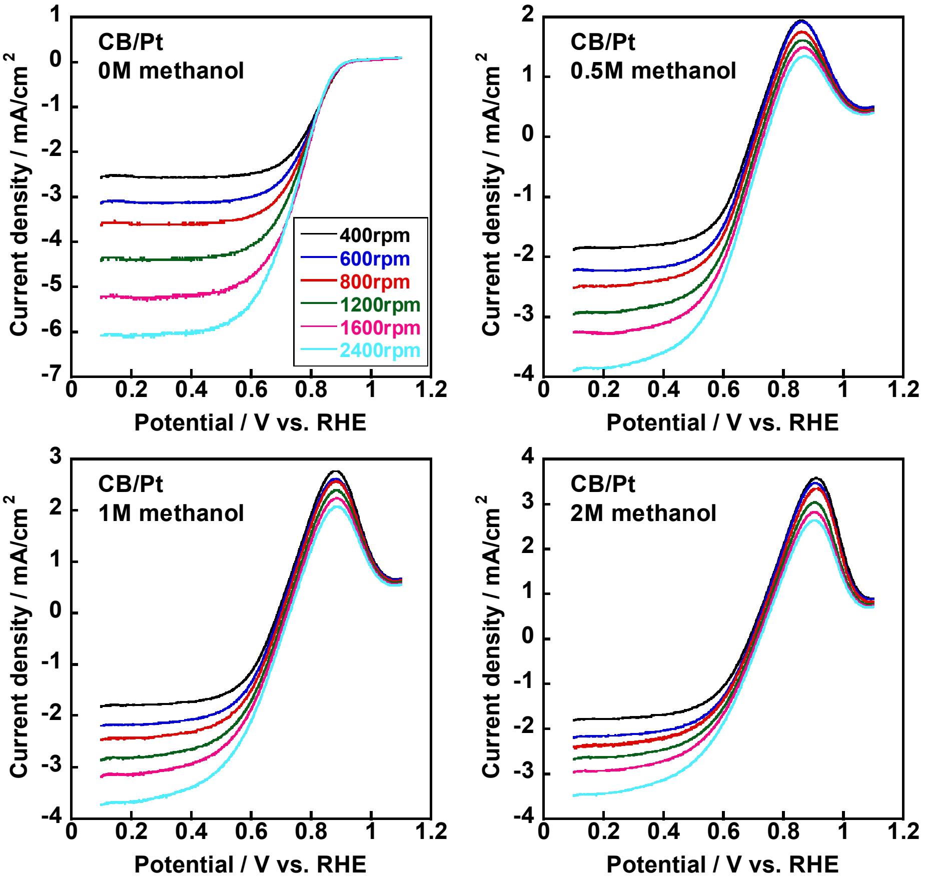

Figure S4| Rotating disc current density of the CB/Pt in O_2-saturated 0.1M $HClO_4$ solutions containing a specified concentration of methanol at 25 ˚C.

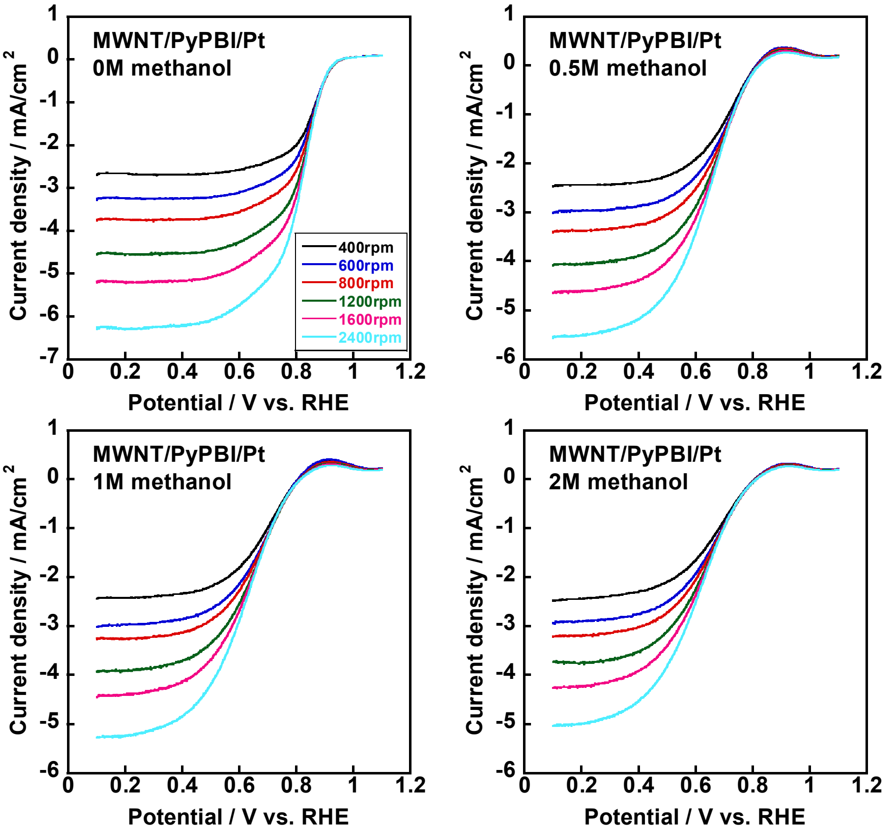

Figure S5| Rotating disc current density of the MWNT/PyPBI/Pt in O_2-saturated 0.1M $HClO_4$ solutions in the presence of specified concentration of methanol at 25 ˚C.

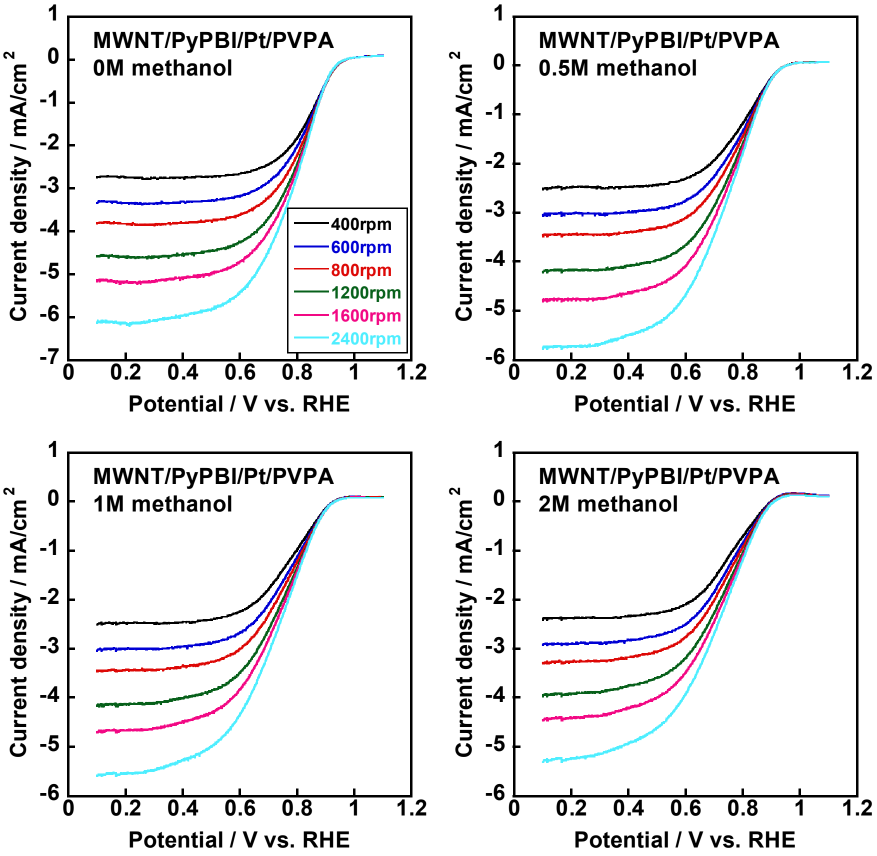

Figure S6| Rotating disc current density of MWNT/PyPBI/Pt/PVPA in O_2-saturated 0.1M $HClO_4$ and in the presence of different concentration of methanol electrolyte at 25 ˚C.

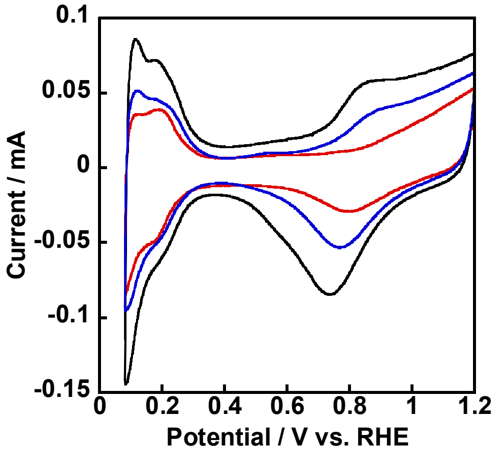

Figure S7| CV curves recorded in 0.1M $HClO_4$ solutions at the scan rate of 50 mV/s for the CB/Pt (black), MWNT/PyPBI/Pt (blue) and MWNT/PyPBI/Pt/PVPA (red).

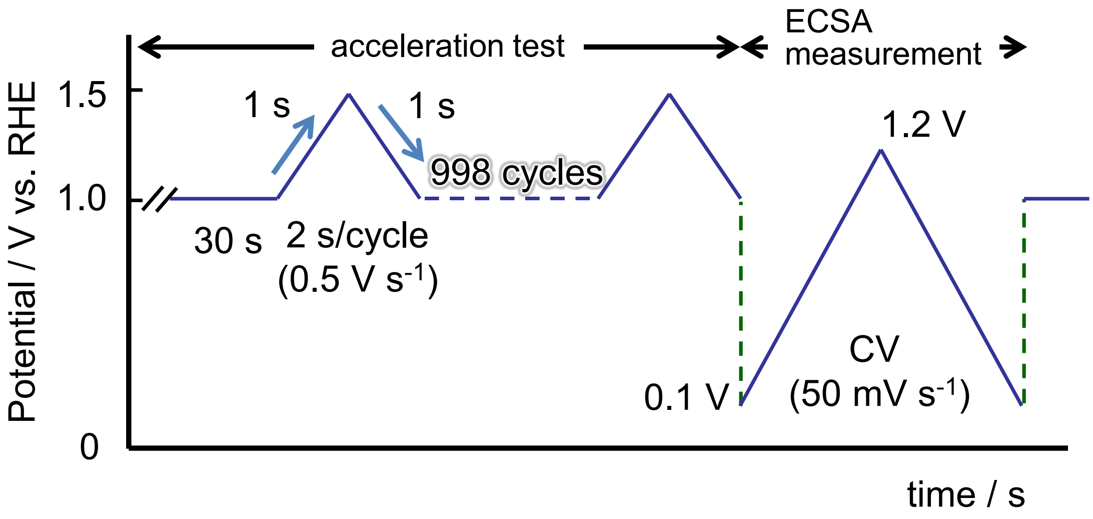

Figure S8| Protocol of durability test for a half-cell based on the Fuel Cell Commercialization Conference of Japan.
